# Coordinated regulation of nitrogen transport and assimilation drives high nitrogen use efficiency in sugarcane

**DOI:** 10.3389/fpls.2026.1813105

**Published:** 2026-06-05

**Authors:** Cui-Xian Qin, Zhong-Liang Chen, Ao-Mei Li, Miao Wang, Jiao-Yun Chen, You-Qiang Pan, Fen Liao, Dong-Liang Huang

**Affiliations:** Guangxi Key Laboratory of Sugarcane Genetic Improvement, Key Laboratory of Sugarcane Biotechnology and Genetic Improvement (Guangxi), Sugarcane Research Institute, Guangxi Academy of Agricultural Sciences, Nanning, China

**Keywords:** glutamate synthase (GOGAT), glutamine synthetase (GS), nitrate transporter (NRT), nitrogen metabolism, sugarcane

## Abstract

Improving nitrogen use efficiency (NUE) is essential for sustainable sugarcane production. Breeding high-NUE varieties enables high yields under low nitrogen (N) input, and identifying NUE-related genes is a critical step for molecular breeding. In this study, we compared two sugarcane genotypes, AS108 (high NUE) and GT11 (low NUE), under normal N (NN) and low N (LN) conditions. AS108 maintained significantly higher biomass, nitrogen content, and NUE under nitrogen limitation. Integrative analyses of enzyme activity, gene expression, and correlation networks indicated that its superior performance results from coordinated, organ-specific enhancement of nitrogen transport and assimilation. AS108 displayed consistently higher Glutamine synthetasec (GS) and glutamate synthase (GOGAT) activities, earlier and stronger induction of high-affinity transporters NRT2 and NRT2.2, and dynamic network reorganization under low nitrogen conditions. Network analysis identified NRT2/NRT2.2 as central hubs coupling nitrate uptake with GS/GOGAT-mediated assimilation. Notably, key nitrate transporter genes peaked 30–60 days earlier in AS108, highlighting the importance of temporal coordination. Together, these findings define a systems-level mechanism in which early-activated nitrate transport drives downstream assimilation to sustain growth under nitrogen limitation, providing molecular targets for breeding high NUE sugarcane cultivars.

## 1 Introduction

Nitrogen (N) is an essential macronutrient and a fundamental determinant of plant growth, productivity, and crop yield (Li et al., 2025). As a structural component of amino acids, nucleic acids, chlorophyll, and numerous cofactors, N directly influences photosynthetic capacity, biomass accumulation, and metabolic homeostasis (Xu et al., 2012). Modern agricultural systems rely heavily on N fertilization to sustain high yields (Masclaux-Daubresse et al., 2010). However, excessive N application has led to severe environmental consequences, including nitrate leaching, eutrophication, soil acidification, and greenhouse gas emissions (Ahmed et al., 2017; Qasim et al., 2021). Improving N use efficiency (NUE) in crops is therefore a central objective for sustainable agriculture, particularly in high-input crops such as sugarcane (Ávila Sáez et al., 2012; Swarbreck et al., 2019; Ali et al., 2025).

Sugarcane (*Saccharum* spp.) is one of the most important industrial crops worldwide, providing nearly 80% of global sugar production and serving as a major feedstock for bioethanol and bioenergy industries (Verma et al., 2025; Wang et al., 2026). Due to its high biomass productivity and long growth cycle, sugarcane has a substantial N demand. N deficiency frequently limits sugarcane yield and sucrose accumulation, whereas excessive N fertilization may delay maturation, reduce sucrose content, and increase production costs (Desalegn et al., 2023; de Castro et al., 2023). Consequently, enhancing NUE while maintaining yield and sugar quality represents a critical breeding target in sugarcane improvement programs.

NUE can be broadly divided into N uptake efficiency (NUpE) and N utilization efficiency (NUtE) (Lupini et al., 2021; Leal et al., 2025). NUpE refers to the plant’s ability to acquire N from the soil, whereas NUtE reflects the efficiency with which absorbed N is converted into biomass and yield. These processes depend on coordinated regulation of nitrate transport, nitrate reduction, ammonium assimilation, and N remobilization (Liu et al., 2025). Understanding the physiological and molecular mechanisms underlying genotypic variation in NUE is essential for developing low-input, environmentally sustainable cultivars.

In aerobic soils, nitrate (NO_3_^-^) is the predominant form of inorganic N available to plants. Nitrate uptake across root cell membranes is mediated by nitrate transporter (NRT) families, which include low-affinity transporters (*NRT1/NPF f*amily) and high-affinity transporters (*NRT2* family) (Orsel et al., 2002; O'Brien et al., 2016). Among these, *NRT1.1* (also known as CHL1 or NPF6.3) functions as a dual-affinity transporter and nitrate sensor, playing a central role in nitrate signaling and root architecture modulation (Sun and Zheng, 2015; Wang et al., 2018; Maghiaoui et al., 2020). High-affinity *NRT2* transporters are particularly important under low-nitrate conditions and are tightly regulated by external N availability and internal N status (Wang et al., 2019; Lima et al., 2022). Differential expression and regulation of *NRT* genes have been associated with enhanced N acquisition and stress tolerance in multiple crops, including rice, wheat, and maize (O'Brien et al., 2016; Wang et al., 2018). However, the functional characterization of nitrate transporter genes in sugarcane remains limited, especially regarding their role in genotypic differences in N efficiency (Leal et al, 2025).

Following uptake, nitrate is reduced to nitrite by nitrate reductase (NR) and subsequently to ammonium by nitrite reductase (NiR) in plastids. The ammonium generated from nitrate reduction or photorespiration is rapidly assimilated into organic forms via the glutamine synthetase/glutamate synthase (GS/GOGAT) cycle, which represents the primary pathway of ammonium assimilation in higher plants (Liu et al., 2022). Glutamine synthetase (GS) catalyzes the ATP-dependent incorporation of ammonium into glutamine, and glutamate synthase (GOGAT) transfers the amide group of glutamine to 2-oxoglutarate to form glutamate (Bernard and Habash, 2009; Liu et al., 2022). This pathway integrates N metabolism with carbon skeleton supply and serves as a key regulatory node in NUE.

Plant genomes typically encode multiple GS isoforms, including cytosolic *GS1* and plastidic *GS2*. *GS2* primarily functions in chloroplasts to reassimilate ammonium released during photorespiration, while *GS1* isoforms are involved in primary ammonium assimilation, N remobilization, and long-distance transport (Mondal et al., 2021; Liu et al., 2022). GS overexpression has been associated with improved NUE and yield stability in several plants, such as tobacco, rice, wheat and barley (Oliveira et al., 2002; Brauer et al., 2011; Hu et al., 2018; Gao et al., 2019). Despite its agronomic importance, the organ-specific regulation and functional differentiation of GS isoforms in sugarcane under varying N regimes remain poorly understood.

N metabolism is highly dynamic and exhibits strong tissue specificity. Roots are primarily responsible for nitrate uptake and initial assimilation, while leaves integrate N assimilation with photosynthesis and photorespiration. Shoots, particularly in sugarcane, function as major carbon sinks and storage tissues, requiring coordinated N allocation to sustain growth and sucrose accumulation. Increasing evidence suggests that efficient NUE depends not only on the expression level of individual genes but also on the coordination among transporters, assimilation enzymes, and developmental cues (Ávila Sáez et al., 2012; Xu et al., 2012; Koltun et al., 2022; Maniero et al., 2023). Systems-level approaches have revealed that N limitation triggers extensive transcriptional reprogramming and network reconfiguration, enabling plants to optimize resource allocation under stress conditions.

Genotypic variation in N response offers valuable opportunities for dissecting the molecular mechanisms underlying NUE. In several crops, low-N-tolerant genotypes exhibit higher nitrate uptake capacity, enhanced GS/GOGAT activity, and earlier activation of N-responsive genes (Han et al., 2015; Li et al., 2021). However, in sugarcane, comprehensive analyses integrating growth performance, enzymatic activity, gene expression, and network relationships across organs and developmental stages remain scarce. Given the complex polyploid genome of sugarcane and its long growth cycle, identifying robust physiological and molecular markers associated with NUE is particularly challenging.

In this study, we investigated the physiological and molecular mechanisms underlying low-N tolerance in two sugarcane genotypes, AS108 and GT11, which exhibit contrasting N responses. We conducted a comprehensive analysis that included: (i) evaluation of growth performance, N accumulation, and NUE under normal and low N conditions; (ii) measurement of GS and GOGAT activities in leaves, shoots, and roots at various developmental stages; (iii) expression profiling of cytosolic *GS1* family members and plastidic *GS2*; (iv) expression analysis of key nitrate transporter genes (*NRT1*and *NRT2*); and (v) correlation network analysis to examine interactions among nitrate transporters, N assimilation enzymes, and gene expression patterns.

By integrating physiological, biochemical, and transcriptional data, we aimed to clarify the regulatory mechanisms responsible for enhanced NUE in AS108 and to identify key molecular components associated with N-efficient traits. A deeper understanding of organ-specific N metabolism and its dynamic regulation under different N supplies will provide valuable insights for sugarcane breeding and support the development of cultivars suited to low-input and environmentally sustainable agricultural systems.

## 2 Materials and methods

### 2.1 Experimental design

The experiment was conducted in a greenhouse at the Sugarcane Research Institute, Guangxi Academy of Agricultural Sciences, Nanning, Guangxi, China (22.84°N, 108.25°E). The greenhouse temperature was maintained at 22-36 °C under natural sunlight.

Two materials were selected from 58 sugarcane accessions based on significant differences in nitrogen use rate: GXASF1-08-11 (AS108, high NUE) and GT11 (low NUE). These contrasting materials were chosen to facilitate the investigation of physiological and molecular mechanisms underlying responses to nitrogen stress. In the initial screening, 58 genotypes were evaluated under low N (LN, 0.2 mM) and normal N (NN, 4 mM) conditions. Under both treatments, GT11 showed NUE values below the overall mean (LN: 134.46 mg DW·mg N^-1^ pot^-1^, representing biomass produced per pot per mg of applied nitrogen; NN: 58.57 mg DW·mg N^-1^ pot^-1^), whereas AS108 exceeded the mean (LN: 195.64 mg DW·mg N^-1^ pot^-1^; NN: 144.43 mg DW·mg N^-1^ pot^-1^).Specifically, AS108 exhibited 1.46-fold and 2.47-fold higher NUE than GT11 under LN and NN conditions, respectively. Detailed screening results were reported previously (Anas et al., 2021a).

Sugarcane seed stalks were cut into 5-cm single-bud setts and germinated in sand under greenhouse conditions (35-38 °C) for 2–3 days. After sprouting, seedlings were grown in the greenhouse. When the first true leaf emerged, uniformly developed seedlings were selected and transplanted into PVC pots (22 cm diameter, 14 cm height), with one plant per pot. To enable the drainage of excess liquid, all pots were perforated at the base and equipped with individual saucers. Each genotype under each N treatment included six biological replicates (n = 6). At sampling, three plants with uniform growth were selected as biological replicates for data collection.

The growth medium consisted of perlite and sand mixed at a 1:3 ratio. Plants were watered every other day until the development of three true leaves, after which N treatments were initiated. N levels and nutrient solution composition were based on Robinson et al. (2008). The full composition of the nutrient solution was as follows: 100 μmol/L Fe-EDTA, 10 μmol/L MnSO_4_, 10 μmol/L H_3_BO_3_, 42.5 μmol/L K_2_HPO_4_, 5 mmol/L K_2_SO_4_, 2.5 μmol/L ZnSO_4_, 2 mmol/L MgSO_4_, 1 μmol/L CuSO_4_, 1 mmol/L CaSO_4_, 0.457 mmol/L KH_2_PO_4_, and 0.35 μmol/L Na_2_MoO_4_. The pH of the solution was adjusted to 5.6. Two treatments were established: normal N (NN, 5 mmol L^-1^ N) and low N (LN, 0.2 mmol L^-1^ N), using NH^4^NO_3_ as the N source. Nutrient solution and distilled water were applied alternately every other day, with 100 mL per pot at each application.

### 2.2 Measurement of sugarcane growth, N content, and N use efficiency

Samples were collected at 30, 60, and 90 days after the initiation of N treatments. Roots, shoots, and leaves were harvested separately, and the biomass of each organ was recorded.

For biochemical and molecular analyses, 1 g of fresh tissue from each organ was immediately frozen in liquid N and stored at -80 °C for subsequent enzyme activity assays and RNA extraction. The remaining plant material was dried at 70 °C to constant weight for N content determination.

N use efficiency (NUE) was calculated as:

NUE = Biomass/Amount of N applied (g DW/g N). Biomass was measured as the dry weight per pot.

### 2.3 Enzyme activity assays

Glutamine synthetase (GS) and glutamate synthase (GOGAT) activities were measured as previously described (Anas et al., 2021b).

Sugarcane tissue was ground into a fine powder in liquid nitrogen, and 0.1 g of the powder was used for enzyme extraction. Subsequently, 1 mL of extraction buffer (containing 50 mM Tris-HCl, 1 mM EDTA, 10 mM β-mercaptoethanol, 1% PVP, and 10% glycerol; pH 8.2) was added. The mixture was centrifuged at 12,800 g for 10 min at 4 °C, and the supernatant (crude enzyme extract) was collected and kept on ice for subsequent assays.

GS activity was determined using the following reaction system: 200 μL crude enzyme extract, 600 μL reaction solution A (80 mM Tris-HCl, 40 mM MgSO^4^, 20 mM glutamate, 20 mM cysteine, and 4 mM EDTA; pH 7.4), and 600 μL reaction solution B (80 mM Tris-HCl, 40 mM MgSO^4^, 20 mM ATP, and 20 mM hydroxylamine). The mixture was incubated at 37 °C for 30 min. Subsequently, 300 μL chromogenic reagent (0.37 M FeCl_3_, 0.67 M HCl, and 0.2 M trichloroacetic acid) was added and the mixture was allowed to stand at room temperature for 10 min. After centrifugation at 5,000 rpm for 5 min at 4 °C, the absorbance of the supernatant was measured at 540 nm. One unit of GS activity was defined as the production of 1 μmol product per hour per gram of tissue.

GOGAT activity was determined using the following reaction mixture: 200 μL crude enzyme extract and 800 μL reaction solution (25 mM Tris-HCl, 10 mM glutamine, 10 mM α-ketoglutaric acid, 1 mM EDTA, and 0.3 mM NADH; pH 7.6). The mixture was gently mixed, and the absorbance at 340 nm was recorded after 20 s and 5 min. One unit of GOGAT activity was defined as the oxidation of 1 nmol NADH per hour per gram of tissue.

### 2.4 RT-qPCR analysis

Total RNA was extracted from sugarcane tissues using a Plant RNA Extraction Kit (Cowin Biotech, Beijing, China) according to the manufacturer’s instructions. RNA concentration and quality were assessed using a Multiskan Sky microplate spectrophotometer (Thermo Fisher Scientific, USA). First-strand cDNA was synthesized using the PrimeScript™ RT Reagent Kit with gDNA Eraser (TaKaRa Biotechnology, Beijing, China) following the manufacturer’s protocol.

Sequences of four glutamine synthetase genes (*ScGS1.a, ScGS1.b, ScGS1.c, ScGS2*), five nitrate transporter genes (*ScNRT1.1A, ScNRT1.1B, ScNRT2.1, ScNRT2.3, ScNRT2.4*), and the reference gene *GAPDH* (EF189713) were retrieved from NCBI for sugarcane and related specie, sorghum,. Gene-specific primers were designed using Primer Premier 5. For each gene, the top 10 primer pairs ranked by software score were selected for preliminary qPCR testing. The final primer pair used in this study was selected based on its stable and relatively high amplification efficiency across different sugarcane organs (**Table 1**).

**Table 1:** RT-qPCR primer sequences for candidate genes.

Gene	NCBI ID	Sequence (Sense5’-3’ )	Product length (bp)
*ScGS1.a*	AY835453	F1: CAACCTCAGCCTCTCGGACA R1: GGCTTTGCTCCTGAGATCCAT	88
*ScGS1.b*	AY835454	F1:ACAAAGCCTGCCTCTACGCTG R1:GGACGGCCCCACTTGGAACT	89
*ScGS1.c*	AY835455	F1: TGCCGATAAGGCATTTGGGC R1:CAACGGACGGCCCCACTTGG	134
*ScGS2*	KY072794	F1: CATCTGAGCTTGTGGGAGA R1: GGGTGAGGAGCGGTAAAT	146
*ScNRT1.1*	OQ434230	F1: GACCAATGCCGCGGCGGAGAC R1: CATGGTGCCCGTGAGGTACGT	183
*ScNRT1.2*	OQ434231	F1: CACGGCGGCGGCCGCGGCGG R1: CGTCGGCCAGGCGCTTCTCC	125
*ScNRT2.1*	XM_002451330	F1: ATCCGCGACAACCTCAACCT R1: GGTGGGCGCCGACAGCATGATG	158
*ScNRT2.3*	OQ434232	F1: TACGACCGCTTCGGCGTCAAG R1: AGAGCCAGTCCGACATGAGG	104
*ScNRT2.4*	OQ434233	F1: TGCTACGGCGTCGGCGGCGCT R1: CCGCGAAGAAGCAGGCGAAG	135
*GAPDH*	EF189713	F1:CTTGCCAAGGTCATCCATG R1:CAGTGATGGCATGAACAGTTG	70

Reverse Transcription-quantitative PCR (RT-qPCR) was performed using TB Green^®^ Premix Ex Taq^T^ II (Tli RNaseH Plus) (TaKaRa Biotechnology, Beijing, China). The procedure followed previously described methods (Ling et al., 2014; Wang et al., 2022, Wang et al., 2025), with three technical replicates per sample.

The mRNA reverse transcription reaction system consisted of 5.0 μL template RNA and 0.5 μL Oligo(dT) primer, with nuclease-free water added to a final volume of 6 μL. The amplification program consisted of an initial denaturation at 95 °C for 2 min, followed by 40 cycles of 95 °C for 5 s, 60 °C for 30 s (fluorescence acquisition), and 72 °C for 20 s. A melt curve analysis was performed from 65 to 95 °C with 1 °C increments to verify amplification specificity.

Relative gene expression levels were calculated using the 2-ΔΔCt method.

ΔCt = Ct (target gene) - Ct (reference gene, GAPDH);

ΔΔCt = ΔCt (sample) - mean ΔCt (control).

### 2.5 Data analysis

All data were statistically analyzed and visualized using Excel 2010 and DPS V19.05 software. Correlation analysis and heatmap visualization were performed using Origin 2025. Intersubject effect analysis (ISEA) was conducted to evaluate the main and interaction effects of genotype, N treatment, growth stage, and tissue type. Statistical significance was set at p ≤ 0.05. Each data point represents the mean of three biological replicates.

## 3 Results

### 3.1 AS108 exhibits enhanced low-N tolerance as reflected by sustained growth and superior N utilization

**Figure 1:**
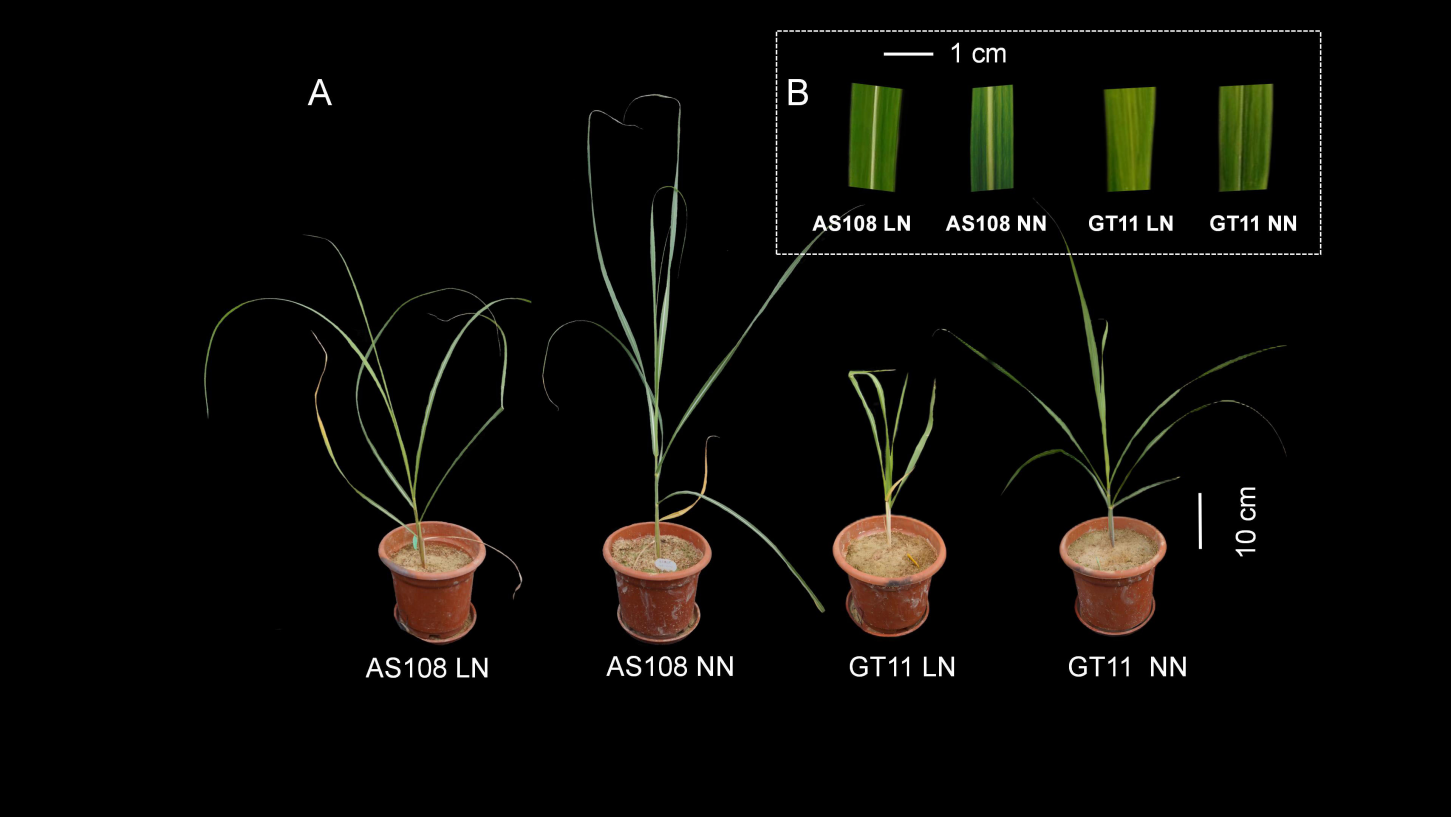
Morphological responses of sugarcane genotypes AS108 and GT11 under different N treatments after 60 days. **(A)** Overall plant phenotype. **(B)** Representative leaf morphology.

From 60 days onward, LN exerted a clear inhibitory effect on the growth of both genotypes. At this stage, AS108 exhibited a stronger low-N tolerance phenotype than GT11, as evidenced by the maintenance of green leaf coloration and normal stem elongation, whereas GT11 leaves appeared yellowish-green (**Figure 1**).

At 90 days after treatment, both genotypes displayed pronounced N deficiency symptoms. Notably, between 60 and 90 days, the total biomass, N accumulation, and N use efficiency of AS108 were 5.68-27.29%, 5.65-45.61%, and 5.69-27.29% higher, respectively, than those of GT11 under both NN and LN treatments (**Table 2**).

**Table 2:** Effects of varying N levels on growth performance and NUE in two sugarcane genotypes.

Treatments		Total biomass (g/plant)		N uptake (mg/plant)		NUE (mg DW/mg N)	
N treatment	Growth stages	AS108	GT11	AS108	GT11	AS108	GT11
LN	30d	2.4	2.8**	0.9	1.0	291.5	335.90**
	60d	6.4**	5.0	3.3**	2.3	380.4**	298.8
	90d	10.6	10.0	4.0*	3.0	420.2**	397.6
NN	30d	2.9	3.1**	1.2	1.2	136.2	166.2
	60d	16.9**	14.3	13.3**	12.6	402.1**	340.2**
	90d	23.9*	21.3	19.4**	17.1	379.2*	337.9

Note: * and ** indicate significant differences between the two genotypes at 30, 60, and 90 days based on single-factor ANOVA. * indicates a significance level of P < 0.05, and ** indicates a significance level of P < 0.01.

### 3.2 Organ-specific regulation of N assimilation enzymes differentiates AS108 from GT11

**Figure 2:**
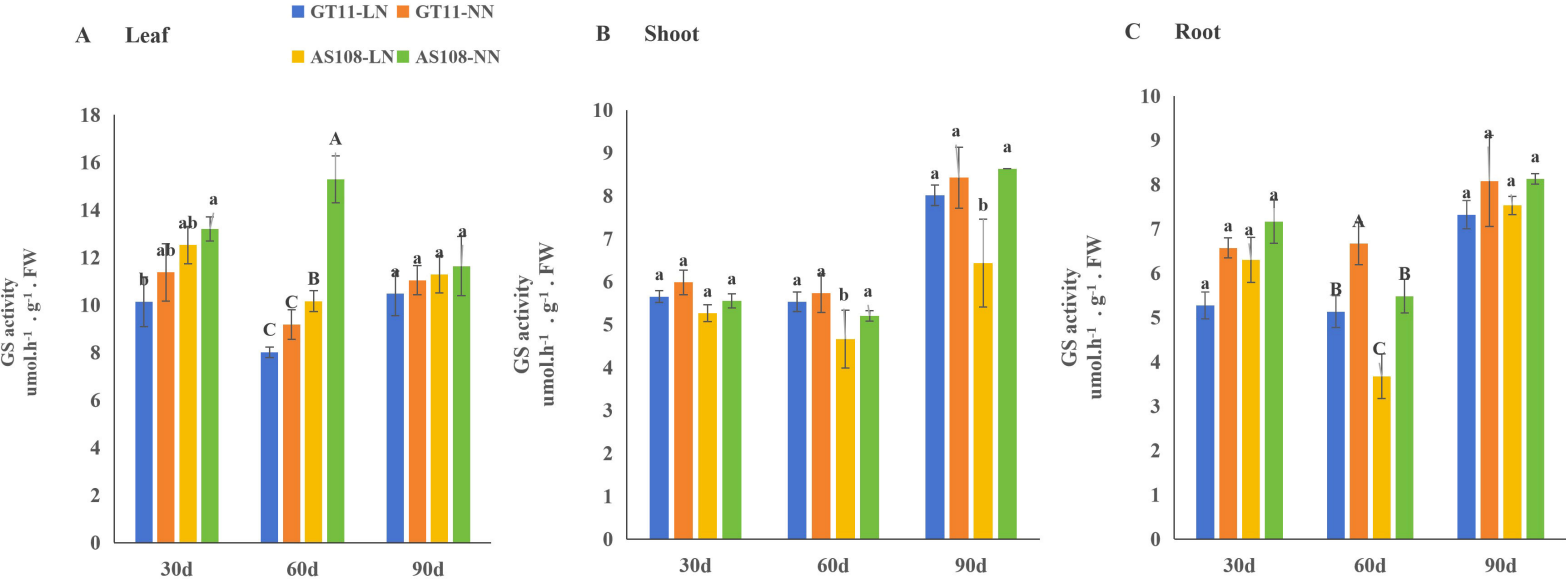
GS activity in leaves, shoots, and roots of two sugarcane genotypes under varying N treatments. **(A–C)** correspond to GS activity in leaves, shoots, and roots, respectively. Capital letters indicate significance at p < 0.01, and lowercase letters indicate significance at p < 0.05. Significance comparisons were made among GT11-LN, GT11-NN, AS108-LN, and AS108-NN.

**Figure 3:**
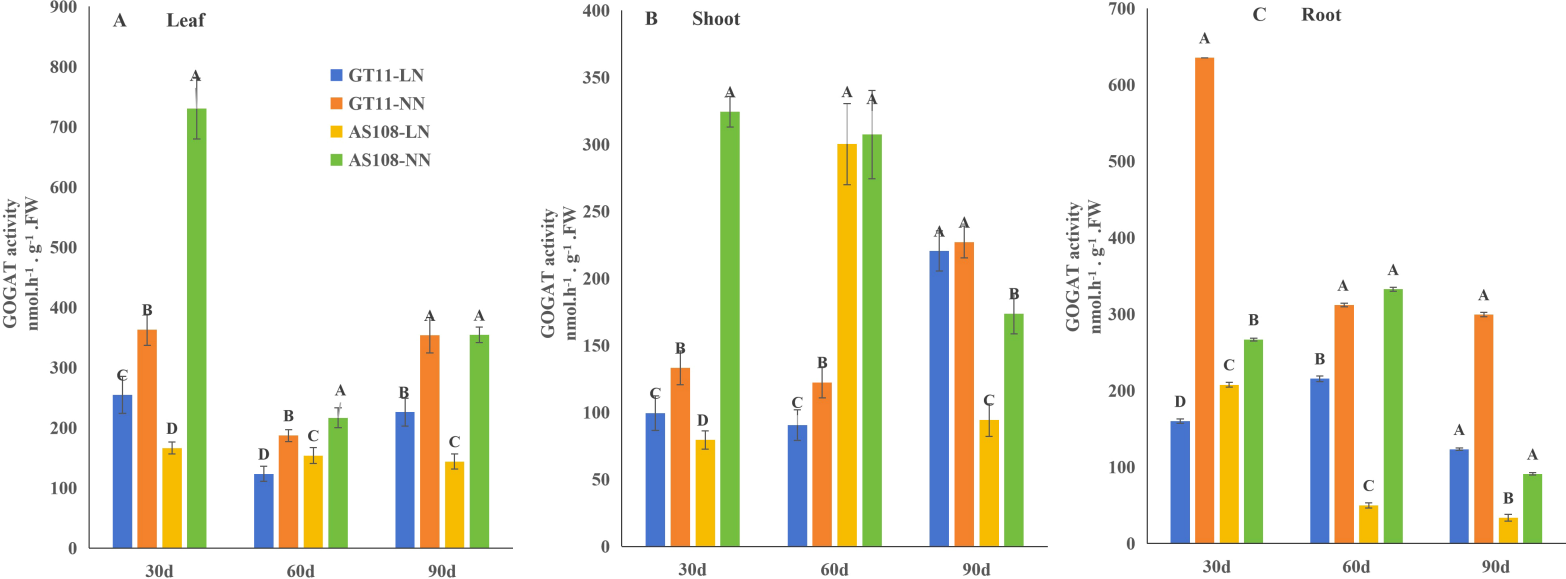
GOGAT activity in leaves, shoots, and roots of two sugarcane genotypes under varying N treatments**. (A–C)** correspond to GS activity in leaves, shoots, and roots, respectively. Capital letters indicate significance at p < 0.01, and lowercase letters indicate significance at p < 0.05. Significance comparisons were made among GT11-LN, GT11-NN, AS108-LN, and AS108-NN.

GS and GOGAT are central enzymes in N metabolism (**Figure 2** & **Figure 3**).Under both N regimes, GS activity in the leaves and roots of AS108 was consistently higher than in GT11 at most growth stages. In leaves, GS activity in AS108 remained relatively high (10.13-15.29 μmol h^-^¹ g^-^¹ FW), exceeding GT11 by 5.34-66.74% between 30 and 90 days after treatment. Notably, under low N (LN) at 60 days, GS activity in AS108 (10.16 μmol h^-^¹ g^-^¹ FW) was 10.80% higher than that of GT11 even under normal N (NN), indicating superior N assimilation capacity under nutrient limitation (**Figure 2A**). In roots, GS activity in AS108 (3.67-8.13 μmol h^-^¹ g^-^¹ FW) was 0.61-19.54% higher than in GT11 at most stages, except at 60 days. Conversely, shoot GS activity in AS108 was consistently 6.90-19.72% lower than in GT11 (**Figure 2C**). These results suggest that the N metabolic advantage of AS108 is tissue-specific, primarily manifested in leaves and roots.

GOGAT activity showed a distinct pattern. In leaves, AS108 exhibited higher activity (143.84-730.16 nmol min^-^¹ g^-^¹ FW), exceeding GT11 by 0.12-101.18% except under LN at 30 days and NN at 90 days. (**Figure 3A**).At 60 days, shoot GOGAT activity in AS108 was 1.5-2.3-fold higher than in GT11(**Figure 3B**). In contrast, root GOGAT activity in AS108 was generally lower than in GT11 throughout most of the experimental period (**Figure 3C**).

Intersubject effects analysis (ISEA) demonstrated that GS and GOGAT activities were significantly influenced by cultivar, N concentration, growth stage, and organ type. GS activity exhibited significant interactive effects among all four factors (P < 0.0001). In contrast, N concentration showed no significant interaction with cultivar, growth stage, or organ in regulating GOGAT activity (P = 0.732, 0.540, and 0.072, respectively; **Supplementary Table 1**).

### 3.3 Upregulated GS1 family expression in roots and shoots contributes to enhanced N assimilation in AS108

**Figure 4:**
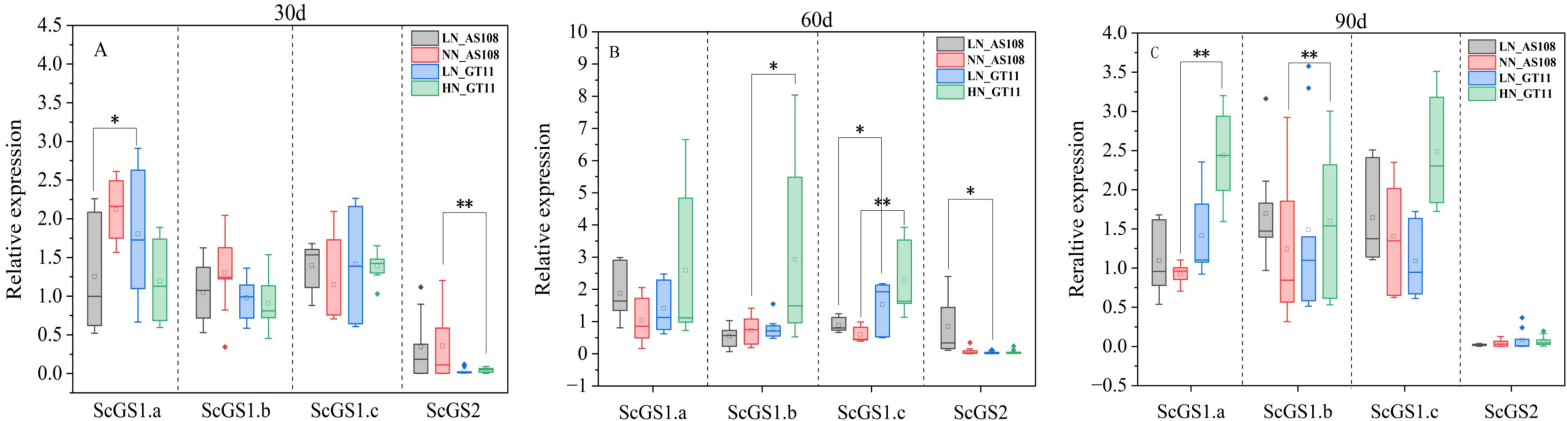
The box plot shows the distribution of the relative expression levels of Sc*GS1.a, GS1.b, GS1.c*, and *ScGS2* gene in different sugarcane genotypes under low- and normal-N treatments. **(A–C)** correspond to the expression of *ScGS1.a, ScGS1.b, ScGS1.c*, and *ScGS2*, respectively, in 30 d, 60 d, and 90 d. * indicates a significance level of P < 0.05, and ** indicates a significance level of P < 0.01.

Members of the cytosolic GS1 gene family (ScGS1.a, ScGS1.b, and GS1.c) and the plastidic ScGS2 gene were expressed in roots, shoots, and leaves of both sugarcane varieties. Overall, GS1 family members exhibited higher expression than GS2 and were more strongly expressed in shoots and roots than in leaves (**Figure 4**; **Supplementary Figure 1**). At 60 days after treatment, when phenotypic differences were most pronounced, ScGS1.a expression in the roots of AS108 under low N was 34.78% higher than in GT11 (1.64 vs. 1.07; **Figure 4B**; **Supplementary Figure 1a1, a3**). Similarly, ScGS1.b expression in AS108 was 1.28- and 1.79-fold higher than in GT11 in leaves and roots, respectively (**Figure 4**; **Supplementary Figures 1b1, b3**). ScGS1.c expression in AS108 reached 1.12, 1.33, and 2.48 in leaves, shoots, and roots, respectively, exceeding GT11 by 20.54%, 106.69%, and 31.71% (**Figures 4A–C**; **Supplementary Figure 1c1-c3**). Across growth stages and N regimes, ScGS2 expression in the leaves and shoots of AS108 was consistently higher than in GT11, ranging from 0.0093- to 97-fold greater (**Supplementary Figure 1 d1, d2**).

ISEA showed that genotype, N treatment, growth stage, and organ type significantly affected the expression of ScGS1.a, ScGS1.b, ScGS1.c, and ScGS2 (**Supplementary Table 1**). However, N concentration had no significant effect on ScGS2 expression and showed no significant interactions with other factors.

### 3.4 Differential regulation of nitrate transporter genes underlies enhanced N uptake in AS108

**Figure 5:**
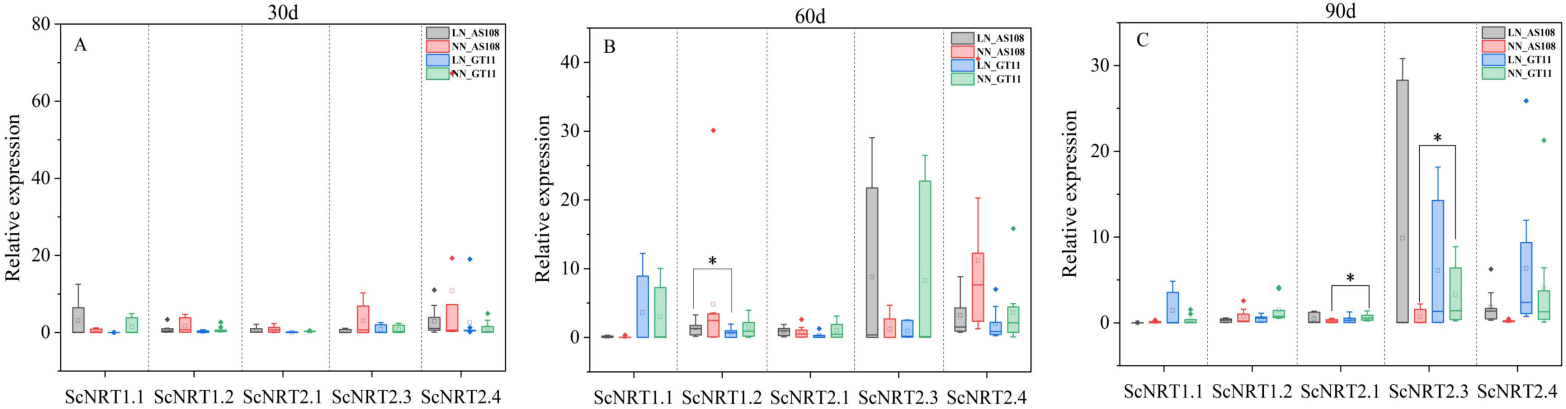
The box plot shows the distribution of the relative expression levels of *ScNRT1.1, ScNRT1.2, ScNRT2.1, ScNRT2.3*, and *ScNRT2.4* gene in two sugarcane genotypes under low- and normal-N treatments. **(A–C)** correspond to the expression of *ScNRT1.1, ScNRT1.2, ScNRT2.1, ScNRT2.3*, and *ScNRT2.4*, respectively, in 30 d, 60 d, and 90 d. * indicates a significance level of P < 0.05, and ** indicates a significance level of P < 0.01.

Nitrate transporters (NRTs) mediate transmembrane nitrate (NO^3-^) transport and are essential for plant N uptake. ScNRT1.1, a dual-affinity transporter, showed negligible expression in leaves and shoots but was strongly expressed in roots. Except under 0.2 mM N at 30 days, root expression in GT11 exceeded that in AS108 (**Figure 5A**; **Supplementary Figure 2a3**). ScNRT1.2, a low-affinity transporter, was mainly expressed in leaves and roots; under low N at 30 and 90 days, leaf expression in AS108 was 5.34- and 2.76-fold higher than in GT11 (**Figures 5A–C**; **Supplementary Figure 2b1**). The high-affinity transporters ScNRT2.1, ScNRT2.3, and ScNRT2.4 displayed distinct patterns. ScNRT2.1 was expressed primarily in leaves and roots, with consistently higher levels in AS108, including 2.65-18.29-fold higher expression in leaves and 49.08-82.93% higher expression in roots; this difference persisted under normal N at 60 days (3.48-fold) (**Figure 5B**; **Supplementary Figures 2c1, c3**). ScNRT2.3 was predominantly root-expressed, with GT11 showing 3.28-288.06% higher root expression than AS108 across treatments and stages (**Supplementary Figure d d1-d3**). ScNRT2.4 was expressed mainly in leaves and roots, and overall expression was higher in AS108; in leaves at 30–60 days, levels were 0.96-51-fold higher, and in roots 10.12-74.36% higher under both N regimes (**Figures 5A,B**; **Supplementary Figures 2 e1-e3**).

ISEA revealed pronounced organ-specific expression patterns among the five analyzed NRT genes. ScNRT1.1 expression was significantly influenced by genotype, N regime, growth stage, and organ type, with significant interaction effects (**Supplementary Table 1**). In contrast, ScNRT2.1 and ScNRT2.3 were mainly regulated by N supply and developmental stage. All five genes exhibited significant expression differences between genotypes under varying N treatments and across organs, displaying strong tissue specificity with predominant expression in roots and leaves and minimal expression in shoots, consistent with roles in root nitrate uptake and leaf nitrate reassimilation. Notably, genotypic variation was most evident for ScNRT1.1, while N treatment and growth stage significantly affected ScNRT1.1, ScNRT2.1, and ScNRT2.3. Peak expression of these key genes occurred earlier in AS108 (30–60 days) than in GT11 (60–90 days) (**Supplementary Figure 2 a1-a3, c1-c3, d1-d3**.

### 3.5 Distinct correlation architectures of N metabolism genes under low and normal N conditions

**Figure 6:**
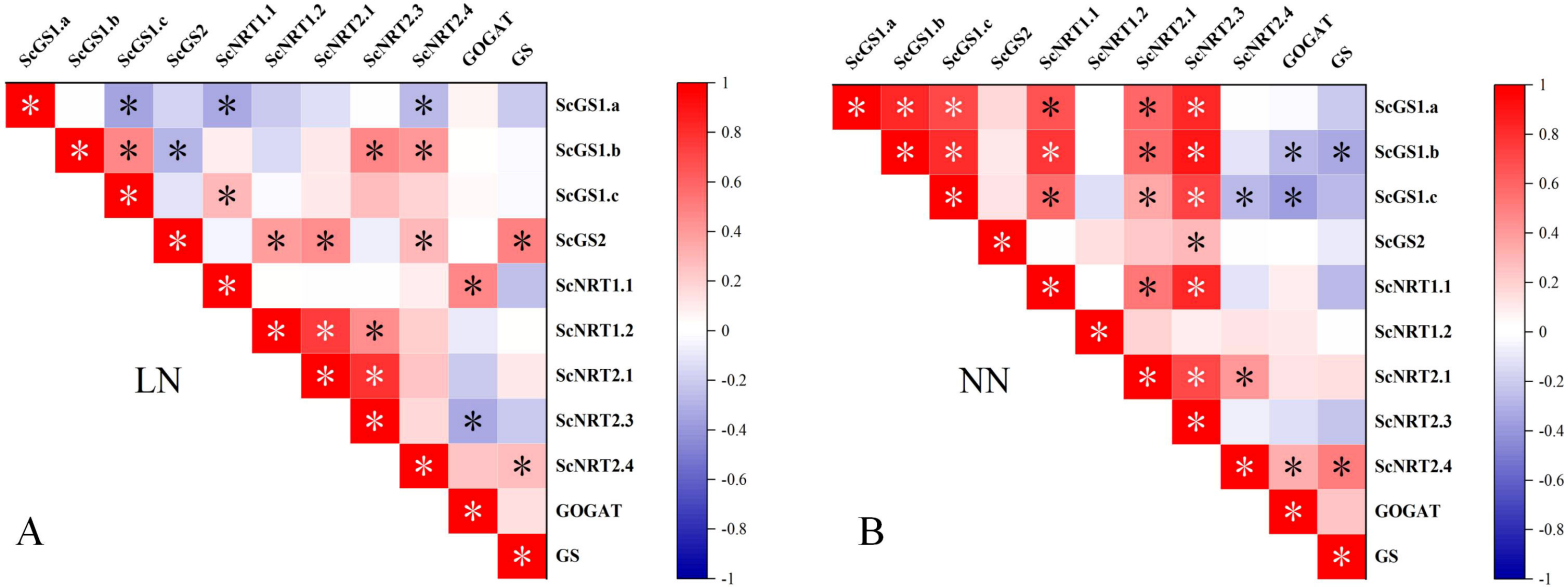
Correlation matrix of four *GS* genes, five *NRT* genes, and GS and GOGAT enzyme activities under low- (LN) and normal-N (NN) conditions. **(A, B)** represent their correlationship under LN and NN treatment, respectively. Blue blocks in the upper right triangle indicate negative correlations, red blocks indicate positive correlations* indicates a significance level of P < 0.05.

To elucidate the relationships among GS and GOGAT enzyme activities and the expression of four GS genes and five NRT genes, a comprehensive correlation analysis was performed (**Figure 6**). The results revealed distinct correlation patterns under LN and NN conditions. Under LN stress, correlations among the four GS genes were predominantly negative. In particular, ScGS2 was significantly negatively correlated with ScGS1.b, whereas ScGS1.b and ScGS1.c exhibited a significant positive correlation (R^2^ = 0.457). Moreover, ScGS2 showed significant positive correlations with ScNRT1.1, ScNRT2.1, and GS enzyme activity. In contrast, under NN conditions, the four GS genes were significantly positively correlated with most NRT genes but negatively correlated with GS and GOGAT enzyme activities.

Across both N regimes, the five NRT genes generally exhibited positive correlations with one another, as well as with the four GS genes and the activities of GS and GOGAT. Notably, ScNRT2.1 and ScNRT2.4 consistently showed significant positive correlations with most GS and NRT genes and with both enzyme activities, suggesting that they may function as central regulatory nodes in the N metabolism network.

## 4 Discussion

N (N) availability is a primary determinant of crop productivity, and improving N use efficiency (NUE) remains a central goal in sustainable sugarcane production (Thorburn et al., 2017; Zhan et al., 2025). In the present study, we demonstrated that AS108 exhibits superior low-N (LN) tolerance compared with GT11, as reflected by sustained biomass accumulation, enhanced N accumulation, higher NUE, and coordinated regulation of nitrate transport and assimilation genes. By integrating physiological, enzymatic, and transcriptional analyses across organs and developmental stages, our results provide a comprehensive view of how genotypic variation in N metabolism underlies differential adaptation to N limitation.

### 4.1 Enhanced growth and N use efficiency under low N

N deficiency typically restricts plant growth by limiting chlorophyll biosynthesis, protein accumulation, and carbon-N metabolic balance (Wen et al., 2020; Xu et al., 2012). In our study, LN treatment inhibited growth in both genotypes after 60 days, yet AS108 maintained greener leaves and normal stem elongation relative to GT11. Between 60 and 90 days, AS108 exhibited 5.68-27.29% greater biomass, 5.65-45.61% higher N accumulation, and 5.69-27.29% higher NUE under both normal N (NN) and LN conditions. These differences indicate that AS108 possesses both constitutive and inducible advantages in N acquisition and utilization.

NUE is generally determined by two major components: N uptake efficiency (NUpE) and N utilization efficiency (NUtE) (Lupini et al., 2021). Our data suggest that AS108 improves both aspects. Higher N accumulation under LN implies enhanced NUpE, while sustained biomass production relative to N content indicates improved NUtE. Similar genotypic variation in NUE has been reported in rice, maize, and wheat, where tolerant varieties maintain growth under N limitation by optimizing N remobilization and metabolic efficiency (Han et al., 2015; Li et al., 2021; Yang et al., 2024). In sugarcane, only one study has reported the physiological and agronomic responses of different varieties to varying N levels (Yang et al., 2019). However, genetic variation in N use efficiency (NUE) remains poorly characterized, highlighting the importance of our findings for breeding cultivars adapted to low-input systems.

### 4.2 Organ-specific regulation of GS/GOGAT supports efficient N assimilation

The GS/GOGAT cycle represents the primary pathway for ammonium assimilation in higher plants (Liu et al., 2022). GS catalyzes the ATP-dependent incorporation of ammonium into glutamine, which is subsequently converted into glutamate by GOGAT. Enhanced GS/GOGAT activity is frequently associated with improved N assimilation and yield stability under stress (Bernard and Habash, 2009; Liu et al., 2022).

In this study, AS108 consistently displayed higher GS activity in leaves and roots than GT11 across most stages and N regimes. Notably, under LN at 60 days, leaf GS activity in AS108 exceeded that of GT11 even under NN, underscoring a robust assimilation capacity independent of external N supply. Elevated leaf GS activity may facilitate rapid reassimilation of photorespiratory ammonium and efficient incorporation of absorbed nitrate-derived ammonium into amino acids, thereby sustaining chlorophyll and protein synthesis under N stress (Avila-Ospina et al., 2015; Mondal et al., 2021; Liu et al., 2022). Enhanced root GS activity further suggests improved assimilation of absorbed nitrate following its reduction to ammonium, and promoting N flux into organic forms.

Interestingly, shoot GS activity in AS108 was consistently lower than in GT11, indicating organ-specific metabolic partitioning. Sugarcane shoots serve as major carbon sinks and storage tissues; reduced GS activity in shoots of AS108 may reflect preferential allocation of N assimilation to source (leaves) and uptake (roots) tissues, thereby optimizing N acquisition and photosynthetic capacity under LN. Organ-specific regulation of GS isoforms has been reported in several plants, where cytosolic *GS1* is primarily involved in N remobilization and transport, whereas plastidic *GS*2 functions in photorespiratory ammonium reassimilation (Mondal et al., 2021).

GOGAT activity showed a more complex pattern. While leaf and shoot GOGAT activities were generally higher in AS108, root GOGAT activity was often lower. Given that GS activity was consistently elevated in AS108 roots, the lower GOGAT activity may reflect dynamic regulation of the GS/GOGAT ratio to balance carbon skeleton availability and N flux. Notably, intersubject effects analysis revealed strong interactive effects of genotype, N concentration, growth stage, and organ type on GS activity, whereas N concentration had limited interaction with other factors for GOGAT. This suggests that GS may represent a primary regulatory control point in N assimilation plasticity, consistent with previous studies identifying GS as a key determinant of NUE (Thomsen et al., 2014).

### 4.3 Upregulation of *GS1* family genes enhances N assimilation capacity

Transcriptional profiling further clarified the molecular basis of enhanced GS activity in AS108. The cytosolic *GS1* family (*ScGS1.a, ScGS1.b, ScGS1.c)* exhibited higher expression levels than plastidic *ScGS2* and was predominantly expressed in roots and shoots. Under LN at 60 days, *ScGS1.a* expression in AS108 roots was 34.78% higher than in GT11, while *ScGS1.b* and *ScGS1.c* showed substantial upregulation across organs.

Cytosolic GS1 isoforms are widely recognized as central regulators of N remobilization and long-distance transport, particularly under N-limited conditions (Bernard and Habash, 2009). Enhanced *GS1* expression in AS108 roots likely promotes efficient assimilation of absorbed nitrate-derived ammonium and facilitates N export to shoots. Upregulation in shoots may support N redistribution toward developing tissues, contributing to sustained growth.

*GS2* expression was consistently higher in AS108 leaves and shoots, although N concentration had no significant effect on its expression. GS2 is primarily localized in chloroplasts and plays a critical role in reassimilating ammonium released during photorespiration (Liu et al., 2022). The maintenance of higher *GS2* expression in AS108 may ensure efficient N recycling within photosynthetic tissues, thereby preserving chlorophyll content and photosynthetic performance under LN, as observed phenotypically.

The significant effects of genotype, N treatment, growth stage, and organ type on *GS* gene expression underscore the complexity of transcriptional regulation in N metabolism. Functional differentiation among *GS* isoforms appears to contribute to the superior adaptive plasticity of AS108.

### 4.4 Coordinated regulation of nitrate transporters facilitates N uptake

N uptake begins with nitrate transport across root membranes, mediated by low- and high-affinity nitrate transporters (NRTs) (O'Brien et al., 2016; Wang et al., 2018). Our results reveal distinct genotypic differences in the expression of *NRT1* and *NRT2* family members.

*ScNRT1.1*, a dual-affinity transporter and nitrate sensor, was predominantly expressed in roots and showed significant genotype-dependent regulation. Although GT11 often displayed higher *ScNRT1.1* root expression, AS108 exhibited earlier peak expression (30–60 days), suggesting a more rapid response to N availability. Early activation of nitrate sensing and uptake pathways may enable AS108 to secure N before severe deficiency develops.

The high-affinity transporters *ScNRT2.1* and *ScNRT2.4* were consistently expressed at higher levels in AS108, particularly in leaves and roots. Under LN, AS108 showed up to 18-fold higher leaf *ScNRT2.1* expression and up to 74% higher root *ScNRT2.4* expression. High-affinity *NRT2* transporters are essential under low external nitrate conditions and are tightly regulated by N status (Orsel et al., 2002; Wang et al., 2018). Their elevated expression in AS108 likely underpins enhanced NUpE under LN.

Interestingly, *ScNRT2.3* expression was higher in GT11 roots across treatments, indicating that not all *NRT* genes contribute equally to improved tolerance. The consistent positive correlations among *ScNRT2.1, NRT2.4*, *GS* genes, and enzyme activities suggest that *ScNRT2.1* and *ScNRT2.4* function as central nodes integrating nitrate uptake with downstream assimilation. Such coordination between uptake and assimilation is crucial to prevent nitrate accumulation and ensure metabolic efficiency (Xu et al., 2012; O'Brien et al., 2016).

In this study, we mainly focused on nitrate-related responses, while the role of NH^4+^ was not examined in detail. One reason is that NH^4+^ can be rapidly converted to NO_3_^-^ in tropical soils through nitrification. Therefore, even when NH^4+^ becomes depleted, NO_3_^-^ can still serve as an effective nitrogen source for plant uptake. Moreover, although sugarcane often shows a preference for NH^4+^, this preference does not necessarily lead to improved nitrogen use efficiency (NUE) (Beatriz et al., 2018; Boschiero et al., 2019). In addition, approximately 10% of nitrogen loss from sugarcane fields occurs through denitrification processes that produce NO^2-^ (Li et al., 2025). Enhancing nitrate uptake and utilization may therefore contribute to improving nitrogen use efficiency in sugarcane. Nevertheless, future studies should also investigate NH^4+^ uptake and utilization, as well as its interaction with nitrate pathways, to better understand the molecular regulatory mechanisms underlying nitrogen absorption and utilization in sugarcane.

### 4.5 Dynamic rewiring of the N metabolism network under low N

Correlation analysis revealed striking differences in network architecture between LN and NN conditions. Under NN, *GS* genes were positively correlated with NRT genes but negatively correlated with enzyme activities, indicating coordinated transcriptional regulation with feedback control at the enzymatic level. In contrast, LN induced predominantly negative correlations among *GS* genes, suggesting functional differentiation and competitive regulation among isoforms.

Notably, *ScGS2* showed significant positive correlations with *ScNRT1.1, ScNRT2.1*, and GS enzyme activity under LN, highlighting its integrative role under stress. This shift toward a more complex and modular network architecture under LN may reflect adaptive reprogramming to optimize N allocation when resources are limited. Similar stress-induced network rewiring has been reported in Arabidopsis and rice, where nutrient deficiency triggers extensive transcriptional reorganization to balance growth and survival (Wang et al., 2018).

The consistent centrality of *ScNRT2.1* and *ScNRT2.4* across N regimes underscores their importance as regulatory hubs. By coordinating nitrate uptake with GS/GOGAT-mediated assimilation, these transporters likely ensure synchronized N flux, contributing to the superior performance of AS108.

### 4.6 Implications for sugarcane breeding and sustainable agriculture

Our findings provide mechanistic insights into the physiological and molecular basis of LN tolerance in sugarcane. The superior performance of AS108 is attributable to (i) enhanced GS activity in leaves and roots, (ii) upregulated *GS1* family expression supporting assimilation and remobilization, (iii) elevated expression of high-affinity nitrate transporters, particularly *ScNRT2.1* and *ScNRT2.4*, and (iv) dynamic reconfiguration of N metabolic networks under stress.

Given the environmental and economic costs of excessive N fertilization, breeding varieties with inherently high NUE is critical for sustainable sugarcane production (Desalegn et al., 2023; de Castro et al., 2023). The coordinated upregulation of nitrate transport and assimilation genes observed in AS108 offers promising molecular markers for breeding programs. Furthermore, the organ-specific regulatory patterns suggest that targeted manipulation of root and leaf N metabolism may yield substantial improvements in NUE.

This study investigated the responses of sugarcane seedlings to nitrogen stress under greenhouse conditions, providing a molecular basis for understanding nitrogen utilization in sugarcane. However, to bridge the gap between theoretical insights and field application, future studies should focus on the middle and late growth stages of sugarcane, which are critical determinants of yield and quality. Furthermore, field experiments incorporating environmental factors such as soil microbial interactions, water stress, and climate variability will be necessary to comprehensively evaluate nitrogen uptake and utilization efficiency and to further validate and refine the regulatory mechanisms identified in this study.

## 5 Conclusion

**Figure 7:**
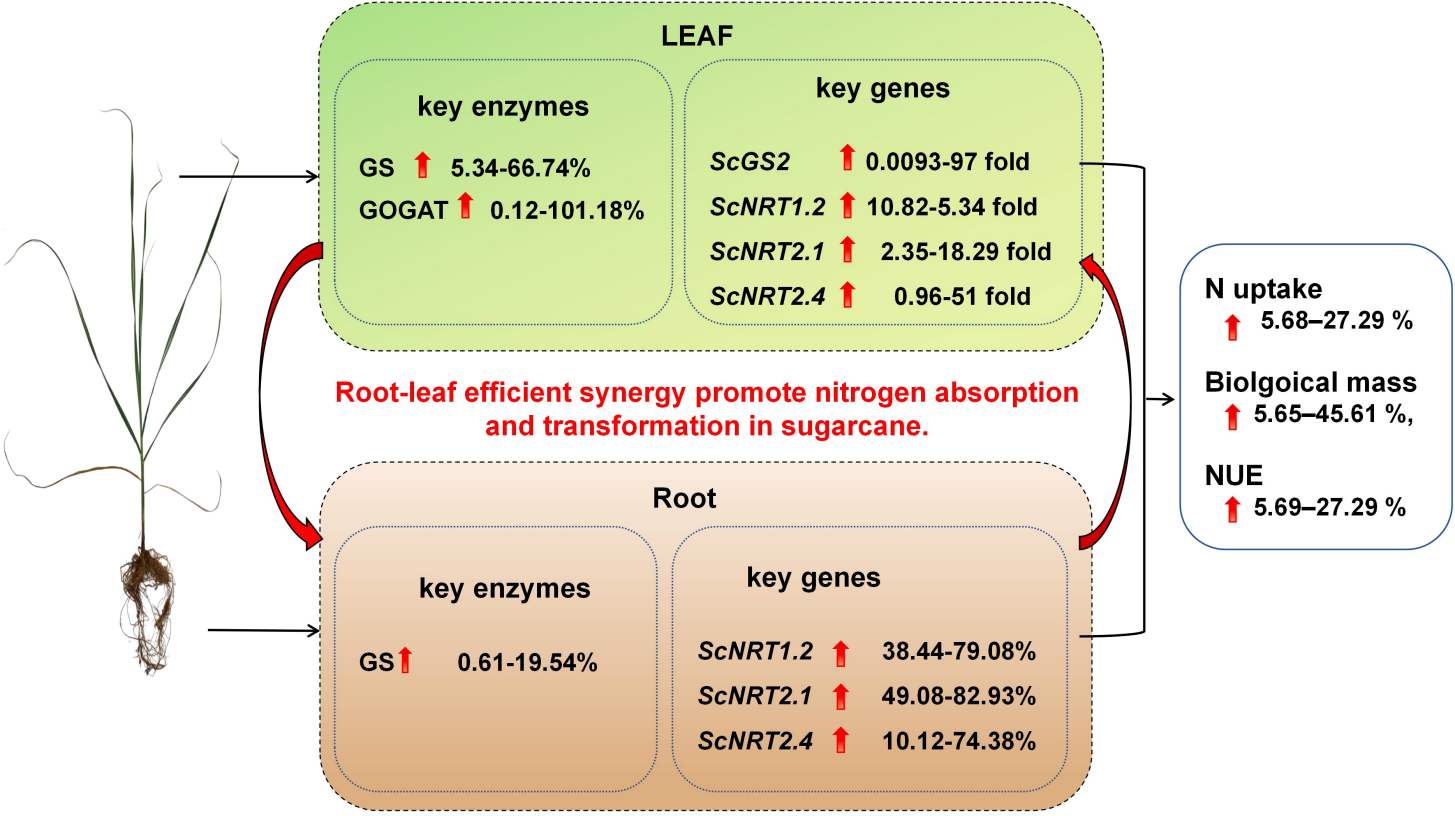
Graphical model of N use efficiency (NUE) mechanisms in sugarcane. The upper green box illustrates up-regulated key enzymes and genes in the leaves of the high-NUE genotype, while the lower light-brown box summarizes corresponding changes in the roots. Red arc arrows connecting the leaf and root sections indicate coordinated interactions between the two tissues. This synergistic up-regulation of key enzymes and genes in both leaves and roots ultimately enhances sugarcane biomass, N accumulation, and N use efficiency.

High NUE genotype exhibits enhanced low-N tolerance through coordinated, organ-specific enhancement of nitrate uptake and assimilation pathways. The integration of physiological performance with molecular regulation highlights a tightly controlled N metabolic network, with *GS1* family members and NRT2 transporters playing central roles. These insights advance our understanding of N efficiency mechanisms in sugarcane and provide valuable targets for breeding cultivars adapted to low-input agricultural systems (**Figure 7**). Future research should focus on the middle and late growth stages of sugarcane and explore genome-wide transcriptional and metabolomic profiling to identify additional regulatory components. Functional validation of key genes, particularly *NRT2* and *GS1* isoforms, through transgenic or gene-editing approaches would clarify causal relationships.

## Data Availability

The original contributions presented in the study are included in the article/**Supplementary Material**. Further inquiries can be directed to the corresponding authors.

## Supplementary material

Supplementary Table 1 Between-subjects effects on GS and GOGAT enzyme activities and the expression of four *GS* genes and five *NRT* genes.Note: P-values are shown. Abbreviations: G, genotype; NT, N treatment; GS, growth stage; O, organ. Interaction terms: G×NT, G×GS, G×O, NT×GS, NT×O, GS×O, interactions between two factors; G×NT×GS, G×NT×O, G×GS×O, NT×GS×O, interactions among three factors; G×NT×GS×O, interaction among four factors. The dark red table indicates that P≤ 0.0001,The red table indicates0.0001≤P ≤0.01,The light red table indicates 0.01≤P ≤0.05,The white table indicates 0.05≤P ≤0.1,The light blue table indicates 0.1≤P ≤0.5,The blue table indicates P ≥ 0.5.

Supplementary Figure 1 Expression levels of ScGS1.a, ScGS1.b, ScGS1.c, and ScGS2 gene in different sugarcane genotypes under low- and normal-N treatments. **(a1-a3, b1-b3, c1-c3, d1-d3)** correspond to the expression of ScGS1.a, ScGS1.b, ScGS1.c, and ScGS2, respectively, in leaves, stems, and roots.

Supplementary Figure 2 Expression levels of *ScNRT1.1, ScNRT1.2, ScNRT2.1, ScNRT2.3*, and *ScNRT2.4* gene in two sugarcane genotypes under low- and normal-N treatments. **(a1-a3, b1-b3, c1-c3, d1-d3, e1-e3)** correspond to the expression of *ScNRT1.1, ScNRT1.2, ScNRT2.1, ScNRT2.3*, and *ScNRT2.4*, respectively, in leaves, stems, and roots.
