# Supplementary material for: Coordinated regulation of nitrogen transport and assimilation drives high nitrogen use efficiency in sugarcane
Source: Front Plant Sci. 2026 Jun 5;17:1813105. doi: 10.3389/fpls.2026.1813105 (PMC13279718; doi:10.3389/fpls.2026.1813105)
Supplement: Supplementary Table 1 — Between-subjects effects on GS and GOGAT enzyme activities and the expression of four GS genes and five NRT genes.Note: P-values are shown. Abbreviations: G, genotype; NT, N treatment; GS, growth stage; O, organ. Interaction terms: G×NT, G×GS, G×O, NT×GS, NT×O, GS×O, interactions between two factors; G×NT×GS, G×NT×O, G×GS×O, NT×GS×O, interactions among three factors; G×NT×GS×O, interaction among four factors. The dark red table indicates that P≤ 0.0001,The red table indicates0.0001≤P ≤0.01,The light red table indicates 0.01≤P ≤0.05,The white table indicates 0.05≤P ≤0.1,The light blue table indicates 0.1≤P ≤0.5,The blue table indicates P ≥ 0.5. [file DataSheet1.docx]

**Table S1. Between-subjects effects on GS and GOGAT enzyme activities and the expression of four *GS* genes and five *NRT* genes**

|  | **Genotypes(G)** | **N Treatment(NT)** | **Growth stage(GS)** | **organ(O)** | **G×NT** | **G×GS** | **G×O** | **NT×O** | **NT×GS** | **O×GS** | **G×NT×GS** | **G×NT×O** | **G×GS×O** | **NT×GS×O** | **G×NT×GS×O** |
| --- | --- | --- | --- | --- | --- | --- | --- | --- | --- | --- | --- | --- | --- | --- | --- |
| **GS** | .001 | <0.001 | <0.001 | <0.001 | <0.001 | <0.001 | <0.001 | <0.001 | <0.001 | <0.001 | <0.001 | .090 | <0.001 | <0.001 | <0.001 |
| **GOGAT** | .009 | <0.001 | <0.001 | <0.001 | .732 | .016 | <0.001 | .544 | .072 | <0.001 | .003 | .008 | <0.001 | .120 | .987 |
| ***ScGS1.a*** | <0.001 | .001 | <0.001 | <0.001 | <0.001 | <0.001 | <0.001 | .009 | <0.001 | <0.001 | <0.001 | <0.001 | <0.001 | <0.001 | <0.001 |
| ***ScGS1.b*** | <0.001 | <0.001 | <0.001 | <0.001 | <0.001 | .001 | <0.001 | <0.001 | <0.001 | <0.001 | <0.001 | .318 | <0.001 | <0.001 | <0.001 |
| ***ScGS1.c*** | <0.001 | <0.001 | <0.001 | <0.001 | <0.001 | <0.001 | <0.001 | <0.001 | <0.001 | <0.001 | <0.001 | <0.001 | <0.001 | <0.001 | <0.001 |
| ***ScGS2*** | <0.001 | .139 | <0.001 | <0.001 | .222 | <0.001 | <0.001 | .002 | .018 | <0.001 | .002 | .004 | <0.001 | .124 | .108 |
| ***ScNRT1.1*** | <0.001 | <0.001 | <0.001 | <0.001 | .001 | <0.001 | <0.001 | .023 | <0.001 | <0.001 | <0.001 | <0.001 | <0.001 | <0.001 | <0.001 |
| ***ScNRT1.2*** | .152 | .069 | .058 | .010 | .472 | .110 | .458 | .395 | .242 | .130 | .365 | .655 | .385 | .575 | .387 |
| ***ScNRT2.1*** | .085 | <0.001 | <0.001 | <0.001 | .009 | .013 | .040 | .055 | .295 | <0.001 | .001 | .020 | .091 | .009 | <0.001 |
| ***ScNRT2.3*** | .360 | <0.001 | <0.001 | <0.001 | <0.001 | .009 | <0.001 | <0.001 | <0.001 | <0.001 | <0.001 | <0.001 | .001 | <0.001 | <0.001 |
| ***ScNRT2.4*** | .227 | .756 | .402 | .001 | .931 | .004 | .011 | .577 | .553 | .058 | .856 | .082 | .003 | .453 | .845 |

Note: P-values are shown. Abbreviations: G, genotype; NT, N treatment; GS, growth stage; O, organ. Interaction terms: G×NT, G×GS, G×O, NT×GS, NT×O, GS×O, interactions between two factors; G×NT×GS, G×NT×O, G×GS×O, NT×GS×O, interactions among three factors; G×NT×GS×O, interaction among four factors.

The dark red table indicates that P≤ 0.0001,The red table indicates0.0001≤P ≤0.01,The light red table indicates 0.01≤P ≤0.05 ,The white table indicates 0.05≤P ≤0.1 ,The light blue table indicates 0.1≤P ≤0.5 ,The blue table indicates P ≥ 0.5 .


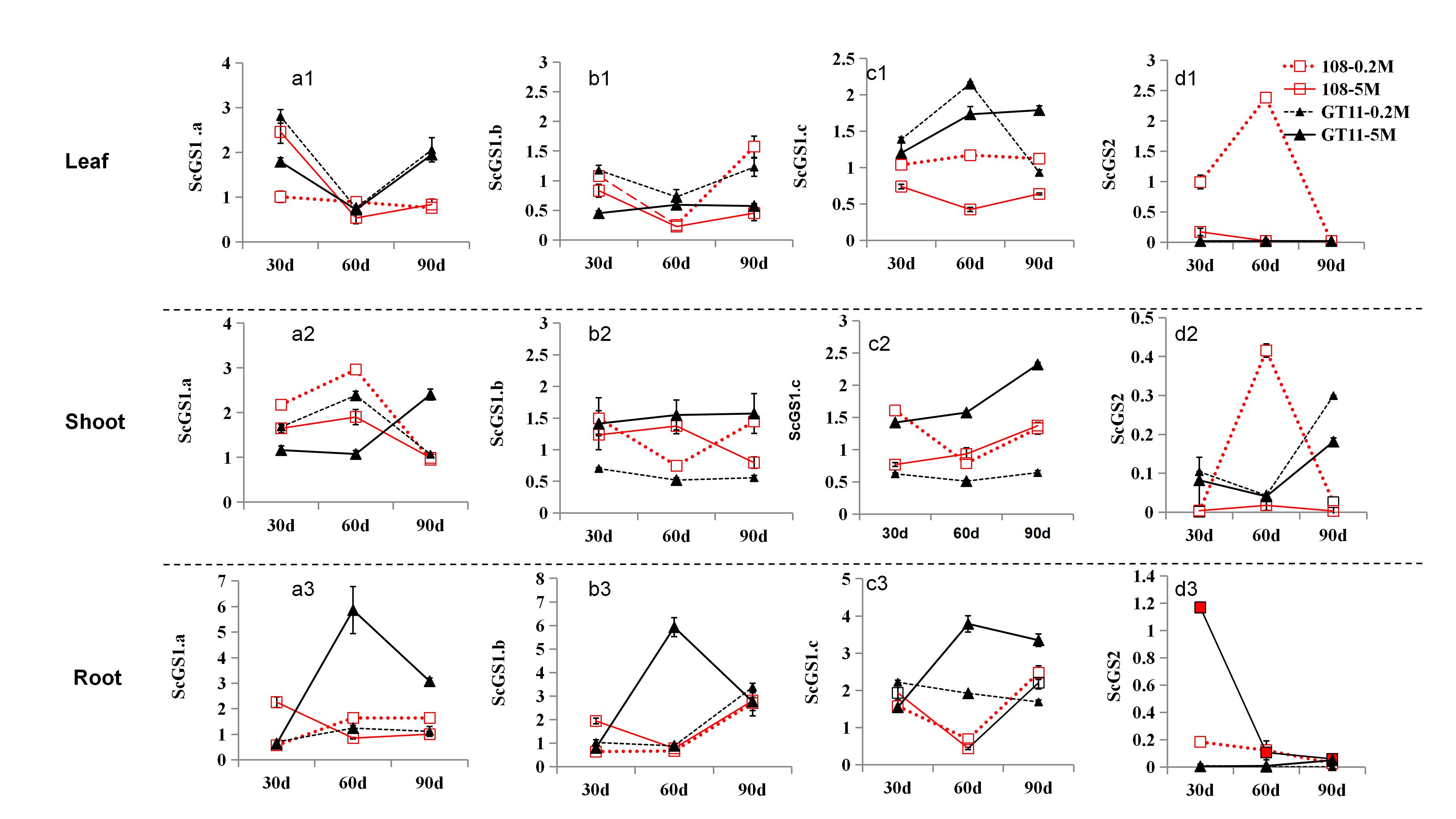


**Figure S1 Expression levels of *ScGS1.a, ScGS1.b, ScGS1.c,* and *ScGS2* gene in different sugarcane genotypes under low- and normal-N treatments.**

Panels a1-a3, b1-b3, c1-c3, and d1-d3 correspond to the expression of *ScGS1.a, ScGS1.b, ScGS1.c,* and *ScGS2*, respectively, in leaves, stems, and roots.


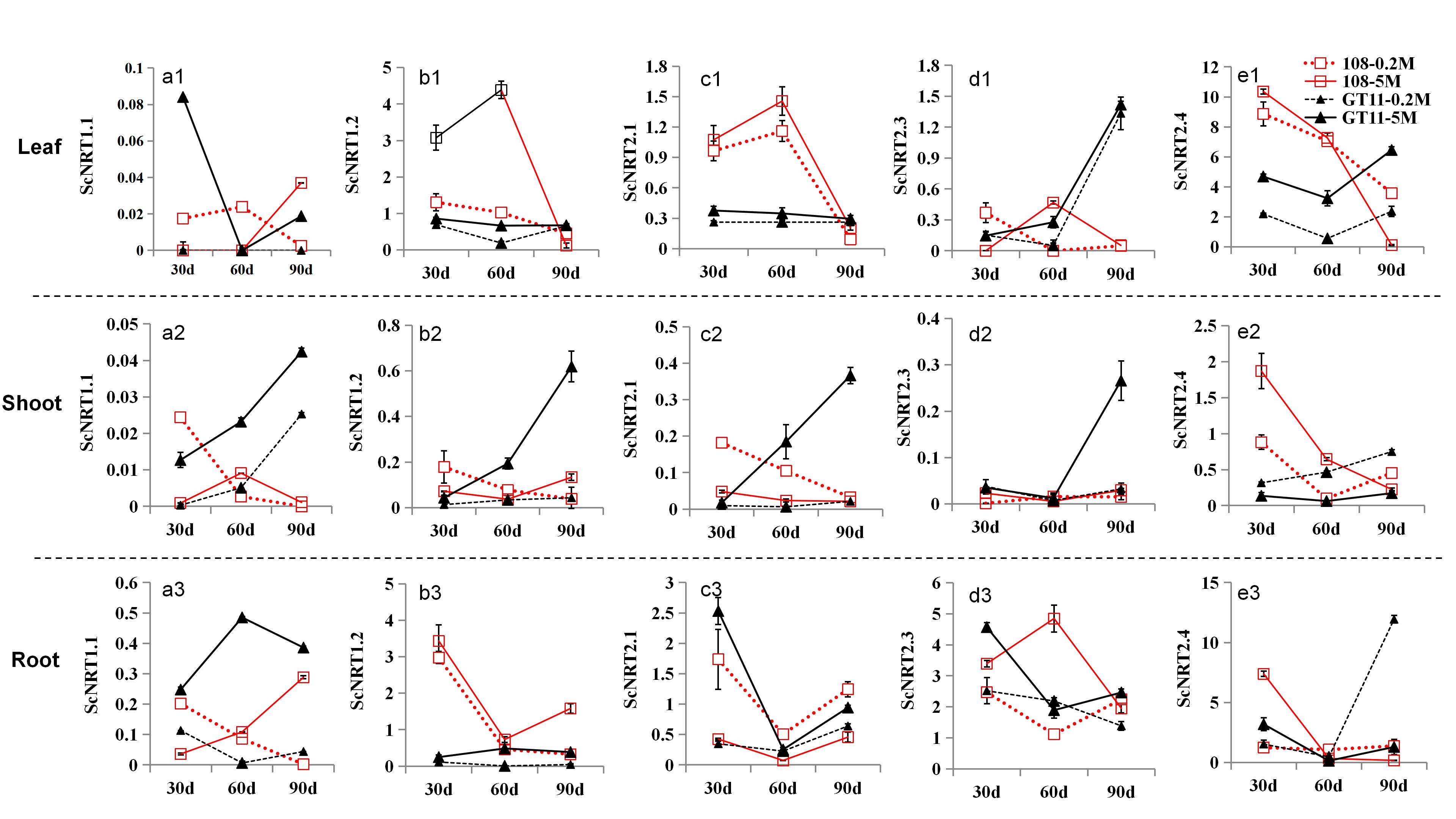


**Figure S2 Expression levels of *ScNRT1.1, ScNRT1.2, ScNRT2.1, ScNRT2.3*, and *ScNRT2.4* gene in two sugarcane genotypes under low- and normal-N treatments.** Panels a1-a3, b1-b3, c1-c3, d1-d3, and e1-e3 correspond to the expression of *ScNRT1.1, ScNRT1.2, ScNRT2.1, ScNRT2.3*, and *ScNRT2.4*, respectively, in leaves, stems, and roots.
